# Complex I Deficiency Due to Selective Loss of Ndufs4 in the Mouse Heart Results in Severe Hypertrophic Cardiomyopathy

**DOI:** 10.1371/journal.pone.0094157

**Published:** 2014-04-04

**Authors:** Edward T. Chouchani, Carmen Methner, Guido Buonincontri, Chou-Hui Hu, Angela Logan, Stephen J. Sawiak, Michael P. Murphy, Thomas Krieg

**Affiliations:** 1 Department of Medicine, University of Cambridge, Cambridge, United Kingdom; 2 MRC Mitochondrial Biology Unit, Cambridge, United Kingdom; 3 Wolfson Brain Imaging Centre, University of Cambridge, United Kingdom; University of Western Ontario, Canada

## Abstract

Mitochondrial complex I, the primary entry point for electrons into the mitochondrial respiratory chain, is both critical for aerobic respiration and a major source of reactive oxygen species. In the heart, chronic dysfunction driving cardiomyopathy is frequently associated with decreased complex I activity, from both genetic and environmental causes. To examine the functional relationship between complex I disruption and cardiac dysfunction we used an established mouse model of mild and chronic complex I inhibition through heart-specific *Ndufs4* gene ablation. Heart-specific *Ndufs4*-null mice had a decrease of ∼50% in complex I activity within the heart, and developed severe hypertrophic cardiomyopathy as assessed by magnetic resonance imaging. The decrease in complex I activity, and associated cardiac dysfunction, occurred absent an increase in mitochondrial hydrogen peroxide levels *in vivo,* accumulation of markers of oxidative damage, induction of apoptosis, or tissue fibrosis. Taken together, these results indicate that diminished complex I activity in the heart alone is sufficient to drive hypertrophic cardiomyopathy independently of alterations in levels of mitochondrial hydrogen peroxide or oxidative damage.

## Introduction

The heart is particularly susceptible to mitochondrial dysfunction due to its strict dependence on aerobic metabolism [1], [2]. Indeed, disruptions in mitochondrial bioenergetics are thought to underlie a variety of chronic myocardial pathologies [3], [4]. However, there are a variety of mechanisms by which mitochondrial dysfunction can drive chronic myocardial pathology. A common feature of mitochondrial dysfunction in the heart is disruption to mitochondrial complex I, caused either by mutation in the genes required to generate a fully functional complex or cumulative damage to the complex itself [5]–[9]. Mitochondrial complex I is the primary entry point for electrons into the mitochondrial respiratory chain and thereby plays an essential role in generating the mitochondrial membrane potential [10], determining the NADH/NAD+ ratio [11], and is also a major source of the reactive oxygen species (ROS) superoxide [12]. Importantly, disruption to all or any of these processes contributes to downstream activation of apoptotic and necrotic cell death pathways [13].

As persistent disruption of complex I activity from a range of causes is frequently observed in cardiomyopathies [5], [8], [9], [14]–[16], we sought to assess directly the consequence of persistent functional disruption of mitochondrial complex I in the heart alone. Disruption to complex I activity could contribute to cell dysfunction through a variety of mechanisms. Dysfunction could be driven by decreasing the mitochondrial membrane potential that would in turn lead to defective ATP synthesis and/or calcium homeostasis [13]. Alternatively, complex I disruption could drive pathology through a buildup of mitochondrial NADH that in turn can drive increased superoxide formation from the flavin site of complex I, or from other sources [12], [17], [18].

To assess the potential primary role of complex I disruption in driving cardiomyopathy through these processes, we chose to disrupt the *Ndufs4* gene, which encodes a 18 kDa subunit of complex I that is not directly involved in electron transport but which plays a role in assembly or stability of the entire complex [19]. *Ndufs4*-null mice have been established as a model for Leigh syndrome; a devastating early onset neurological disorder often associated with mutations to respiratory complexes [19], [20]. These mice exhibit increasing ataxia and failure to thrive early in life, and typically die by post-natal day 55 [19], [20]. In contrast, a recently developed heart specific *Ndufs4*-null mouse strain has no reported pathological phenotype up to 1 year of age despite significant inhibition of complex I activity [21]. Therefore, we employed this heart specific *Ndufs4*-null mouse to explore how persistent inhibition of mitochondrial complex I activity affected cardiac function specifically.

Here we demonstrate that selective disruption of the *Ndufs4* gene in the heart leads to a ∼50% decrease in complex I activity, that in turn drives severe hypertrophic cardiomyopathy, as determined by magnetic resonance imaging (MRI). However, *Ndufs4* gene ablation had no effect on mitochondrial hydrogen peroxide levels, oxidative damage, apoptosis, or tissue fibrosis in the heart. This work suggests that disruption to complex I alone is sufficient to drive myocardial dysfunction. Furthermore, this complex I driven cardiomyopathy occurs through disruption of bioenergetic function and is independent of ROS-dependent pathways.

## Materials and Methods

### Ethics statement

All animal experiments were carried out in accordance with the UK Animals (Scientific Procedures) Act 1986 and the University of Cambridge Animal Welfare Policy, and were approved by the UK Home Office under project license 80/2374.

### Mouse breeding and maintenance

The *Ndufs4-*null mouse model was a kind gift from the lab of Prof Nils Göran-Larsson, Max Planck Institute for Ageing, Cologne, Germany [21].

Previously, these mice were generated by breeding *Ndufs4* ^Loxp/+^ mice with mice carrying the heart specific cre recombinase, CKM-NLS-cre and established on a C57Bl/6 background [22], [23]. Then double heterozygous offspring were crossed to obtain homozygous Ndufs4^LoxP/LoxP^ mice [21]. In order to generate littermate knockout (CKM-NLS-cre; Ndufs4^LoxP/LoxP^) and control mice, the male CKM-NLS-cre knockouts (CKM-NLS-cre; Ndufs4^LoxP/LoxP^) were crossed to control females (Ndufs4^LoxP/LoxP^). Mice with genotype Ndufs4^+/LoxP^ or Ndufs4^LoxP/LoxP^ were used as controls [21]. A mix of CKM-NLS-cre; Ndufs4^LoxP/LoxP^ males and females between the ages of 8–24 weeks were used for all experiments. As described previously, within this age range there was no discernable difference in health, appearance or behaviour between heart-specific *Ndufs4-*null mice and controls.

### Magnetic resonance imaging of heart function

Animals (8–24 weeks of age) were anaesthetized with gaseous isoflurane both for induction (3% in 1 l/min O_2_) and maintenance (1.25–2% in 1 l/min O_2_). A pressure sensor for respiration rate was used to monitor anaesthesia depth, breathing rate was maintained in the range 30–60 breaths per minute. Prospective gating of the MRI sequences was achieved with ECG monitoring. Body temperature was monitored using a rectal thermometer and kept constant at 37°C using a water-heated blanket.

Cine MRI was performed at 4.7 T with a Bruker BioSpec 47/40 system (Bruker Inc., Ettlingen, Germany)[24]. A quadrature birdcage coil of 12 cm was used for signal excitation and a four-channel cardiac receiver coil for signal reception. Animals were positioned prone. After initial localization images, long axis views were acquired. Using these scans as a reference, short axis slices were arranged perpendicularly to both the long-axis views to cover the LV (FISP, TR/TE 6 ms/2.1 ms, 13–20 frames, 3.5 cm FOV, 256×256 matrix, 1 mm slice thickness, bandwidth 78 kHz, flip angle 20°, NEX 1). Full LV coverage was achieved with no slice gap with 8–10 slices.

After the acquisition of the standard protocol, MRI was performed using a multi-slice inversion recovery sequence as described previously.

For image analysis the papillary muscles and trabeculations were excluded from the delineation of the LV at each phase of the cardiac cycle. The regions from each slice were combined using Simpson's rule to provide LV mass, end diastolic volume, end systolic volume, stroke volume and ejection fraction using Segment v1.9 [25].

### Measurement of complex I activity

Mitochondrial complex I activity was assessed as described previously [7]. Briefly, mitochondria were isolated from entire hearts by differential centrifugation [26] and 100 μg mitochondrial protein was resuspended in a 10 mM HEPES, 120 mM KCl assay buffer containing 100 μM NADH, 10 μM decylubiquinone, 300 nM antimycin, 2 mM potassium cyanide, and 30 μg/ml alamethicin and incubated for 5 min at 32°C while the rate of absorbance change was determined at 340 nm. The background rate following addition of 4 μg/ml rotenone was subtracted from the NADH:decylubiquinone rate. In parallel, the citrate synthase activity of each sample was determined as described previously [7]. The complex I rate was normalized to the citrate synthase rate, and then expressed as a % of the appropriate control.

### Measurement of mitochondrial hydrogen peroxide level *in vivo*

Mitochondrial hydrogen peroxide was measured *in vivo* using the MitoB mass spectrometric probe as described previously [7], [27]. Briefly, 75 nmol MitoB (∼3 μmol/kg for 25–30 g mice) in 50 μl saline was administered by tail vein injection to the mouse. 2 hours following injection the entire heart was frozen in liquid nitrogen and then stored at −80°C. For analysis the tissues were homogenized and spiked with deuterated internal standards, extracted, and the content of MitoB and MitoP determined by liquid chromatography and tandem mass spectrometry as described previously [27].

### Measurement of protein carbonylation

Protein carbonylation levels in the heart were determined by rapid homogenization of entire hearts and protein quantification of heart homogenate, followed by quantification of protein carbonyls using the BioCell Protein Carbonyl Assay Kit according to the manufacturers instructions.

### Measurement of apoptosis activation

The expression of caspase 3 and cleaved caspase 3 (Cell Signaling Technologies, 1∶1000 dilution) was analyzed by standard Western blotting techniques. The band intensity of three independent experiments was measured using SigmaGel software and normalized to Actin (Cell Signaling Technologies, 1∶1000 dilution).

### Measurement of MnSOD levels

The expression of manganese superoxide dismutase (MnSOD) (Cell Signaling Technologies, 1∶1000 dilution) was analyzed by standard Western blotting techniques. The band intensity of three independent experiments was measured using SigmaGel software and normalized to Actin (Cell Signaling Technologies, 1∶1000 dilution).

### Histological staining for fibrosis

Hearts were excised, stored overnight in 10% formalin and embedded in paraffin before 9 mircons thick section were taken throughout the heart. Masson's Trichrome staining was performed according to the manufacturers specifications (HT15, St. Louis, MO, USA). Briefly, slides were deparaffinized in deionized water and fixed in Bouin's solution. After washing and incubation in Weigert's Irono Heamatoxylin solution, the slides were incubated in Biebrich Scarlet Acid Fuchsin and Anilin Blue solution for staining. Finally the slides were dehydrated in ethanol and mounted. The nuclei were stained black, the collagen fibers blue and muscles red

### Statistics

Data are presented as mean ± standard error from the mean. Statistical analysis was performed using one-way ANOVA. The differences were considered significant at P<0.05.

## Results and Discussion

**Figure 1:**
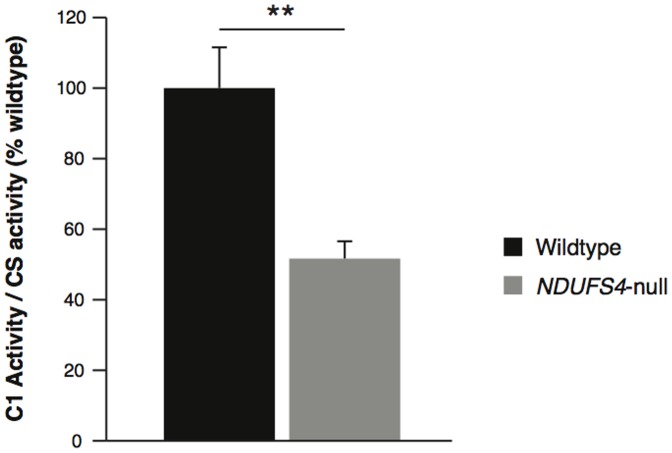
Complex I activity in control and *Ndufs4*-null mouse hearts. Mitochondria isolated from mouse hearts were assessed for rotenone-sensitive NADH:decylubiquinone activity. To control for mitochondrial content from heart isolations, complex I activity was expressed as a function of citrate synthase activity from the same hearts. n = 6, ** p<0.01.

To study how partial disruption of complex I activity impacted on heart function, we used the recently developed heart-specific *Ndufs4*-null mouse model [21]. As expected, *Ndufs4*-null mice exhibited ∼50% lower myocardial complex I activity compared to controls (Fig. 1). This activity of complex I in the *Ndufs4*-null mice was higher than previously reported, which was reported as less than 5% residual activity [21]. This discrepancy is perhaps explained by our use of decylubiquinone as an electron acceptor in contrast to previous assessments which used coenzyme Q_1_ [21], which is a less effective complex I substrate than decylubiquinone [28]. Supporting this interpretation, the previous study also observed a combined complex I/complex III activity in the *Ndufs4*-null mouse that was ∼21% of control levels [21], consistent with their assessment of complex I activity using coenzyme Q_1_ underestimating complex I activity. Therefore, we are confident that the heart selective ablation of the *Ndufs4* gene leads to a decrease in complex I activity of about 50%, similar to that found in a number of pathologies [5], [8], [9], [14]–[16], making this model an effective test for the consequences of complex I disruption on cardiac dysfunction.

**Figure 2:**
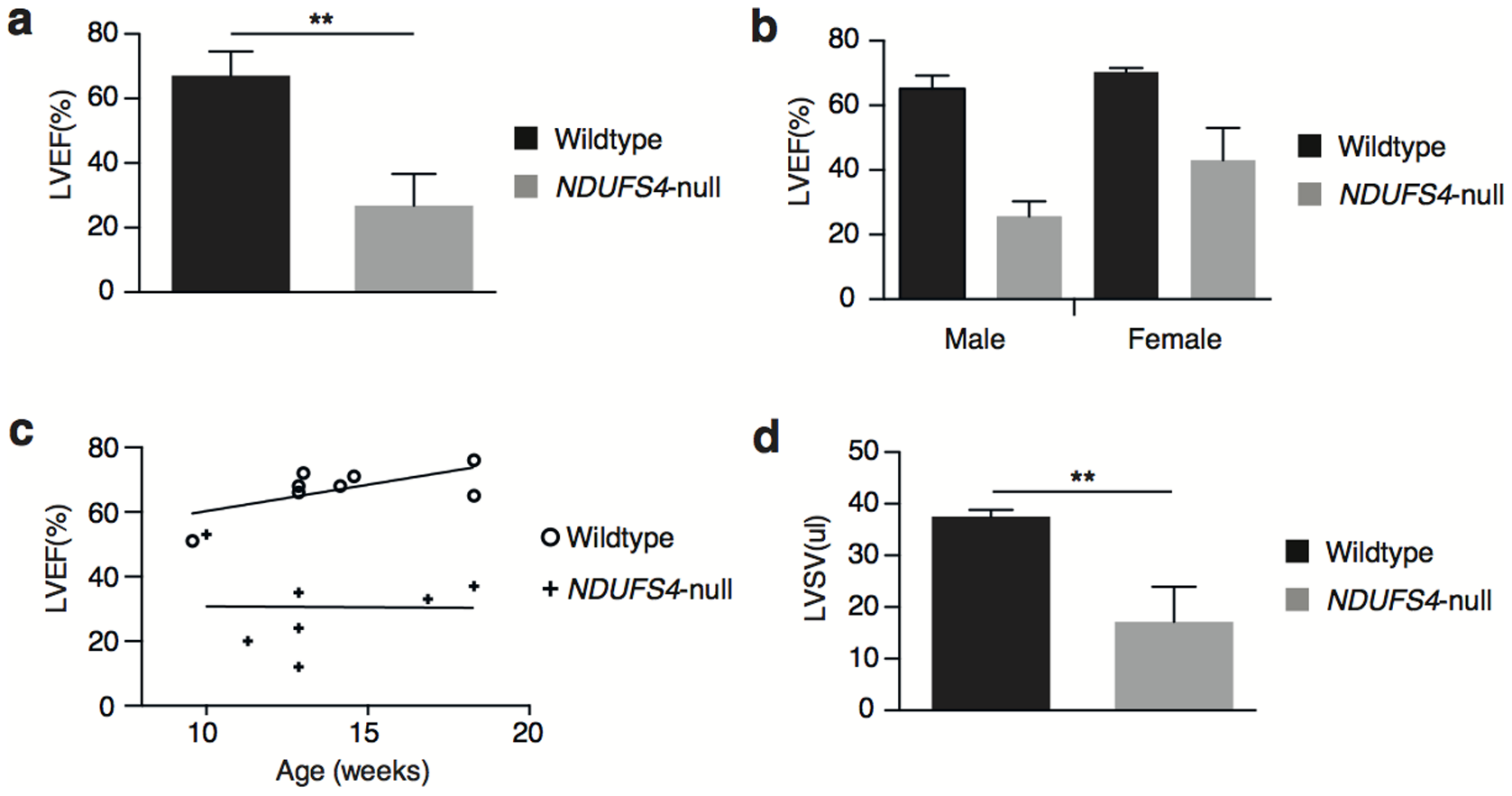
Cardiac function assessed by MRI. a, Left ventricular ejection fraction (LVEF) of control and *Ndufs4*-null mouse hearts assessed *in vivo* by MRI. **b**, LVEF comparison by sex and **c**, age. **d**, left ventricular stroke volume (LVSV) of control and *Ndufs4*-null mouse hearts assessed *in vivo* by MRI. n = 2–8, ** p<0.001.

We next assessed the effects of partial complex I disruption through *Ndufs4* ablation on heart function using MRI (Videos S1 and S2). MRI is the current “gold standard” method for clinical assessment of myocardial function and left ventricular mass, which have been established as sensitive predictors of adverse outcomes in hypertrophic cardiomyopathies [29]. Left ventricular ejection fraction (LVEF) was significantly lower in *Ndufs4*-null mice (26.8%±4.0% versus 67.1%±2.6% in control mice, p<0.001; Fig. 2a). Decreased LVEF was substantial in both male and female mice, with male mice potentially exhibiting a more severe effect (Fig. 2b), although the difference between sexes was not statistically significant. Additionally, the observed difference in LVEF between wild-type and *Ndufs4*-null mice was consistent throughout early life (Fig. 2c) A concomitant decrease in left ventricular stroke volume (LVSV) was also observed in *Ndufs4*-null mice; (17.2 μl±2.8 μl versus 37.50 μl±0.46 μl in control mice, p<0.001; Fig. 2d).

**Figure 3:**
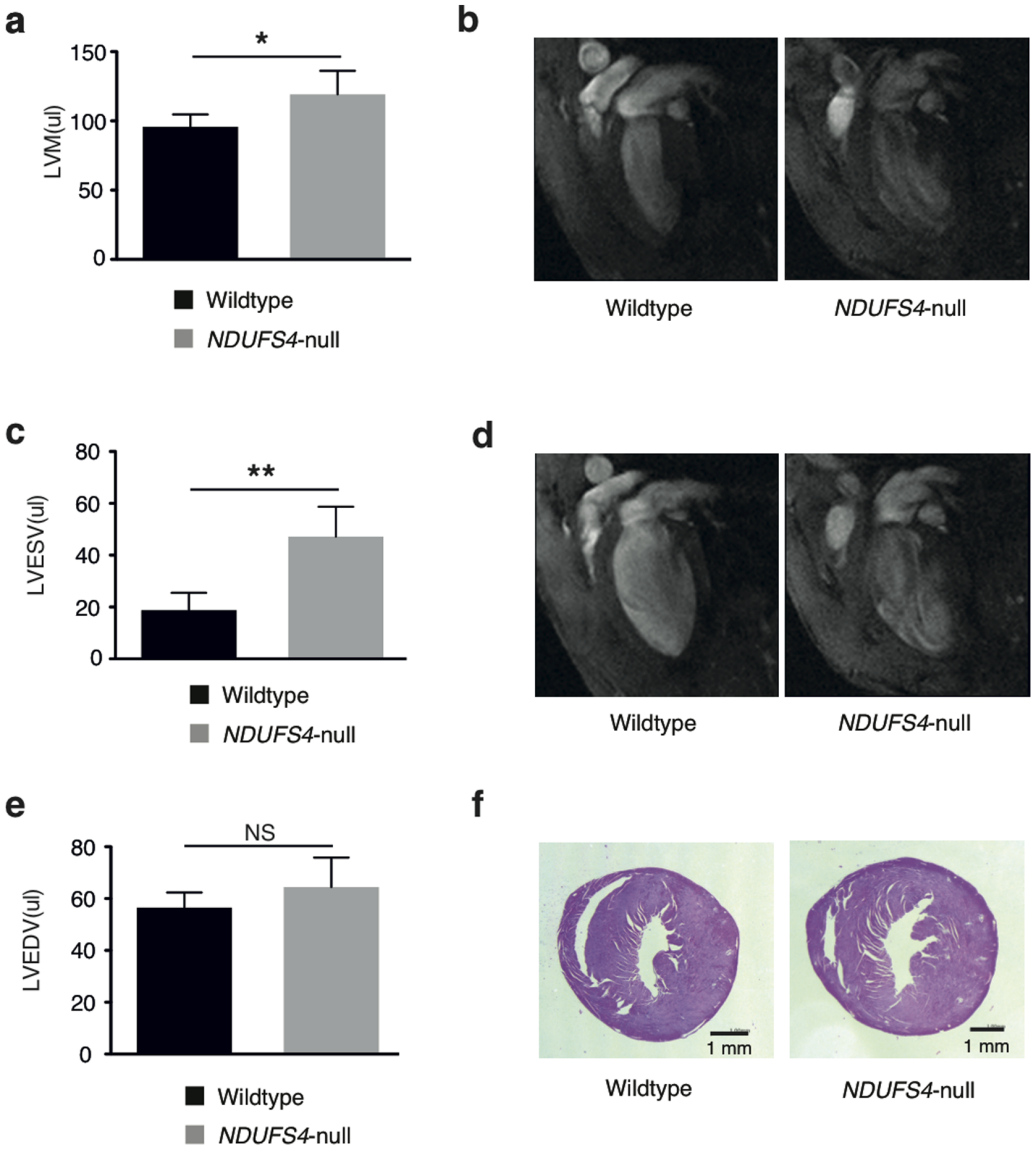
Cardiac morphology assessed by MRI. a, Left ventricular mass (LVM) of control and *Ndufs4*-null mouse hearts. **b**, representative long-axis views of the end systole and **c**, quantification of left ventricular end systolic volume (LVESV) of control and *Ndufs4*-null mouse hearts. **d**, representative long-axis views of the end distole and **e**, left ventricular end diastolic volume (LVEDV) of control and *Ndufs4*-null mouse hearts. All parameters assessed *in vivo* by MRI. **f**, Representative heart slices after Masson trichrome staining of control and *Ndufs4*-null hearts. n = 4–8, * p<0.05, ** p<0.001.

In addition, left ventricular mass (LVM) was significantly higher in *Ndufs4*-null mice (119.0 μl±6.96 μl versus 95.75 μl±3.17 μl in controls, p = 0.005; Fig. 3a). LVM increases were paralleled by a nearly threefold increase in left ventricular end-systolic volume (LVESV, 47.17 μl±4.72 μl versus 18.75 μl±2.38 μl in controls, p<0.001; Fig. 3b and c). Furthermore, there was no significant difference observed in left ventricular end-diastolic volume (LVEDV) between groups (Fig. 3d and e). We next determined whether the severe defects in cardiac function and increase in LVM initiated by *Ndufs4* ablation were accompanied by activation of tissue fibrosis, but found no detectable evidence for fibrotic remodeling (Fig. 3f).

**Figure 4:**
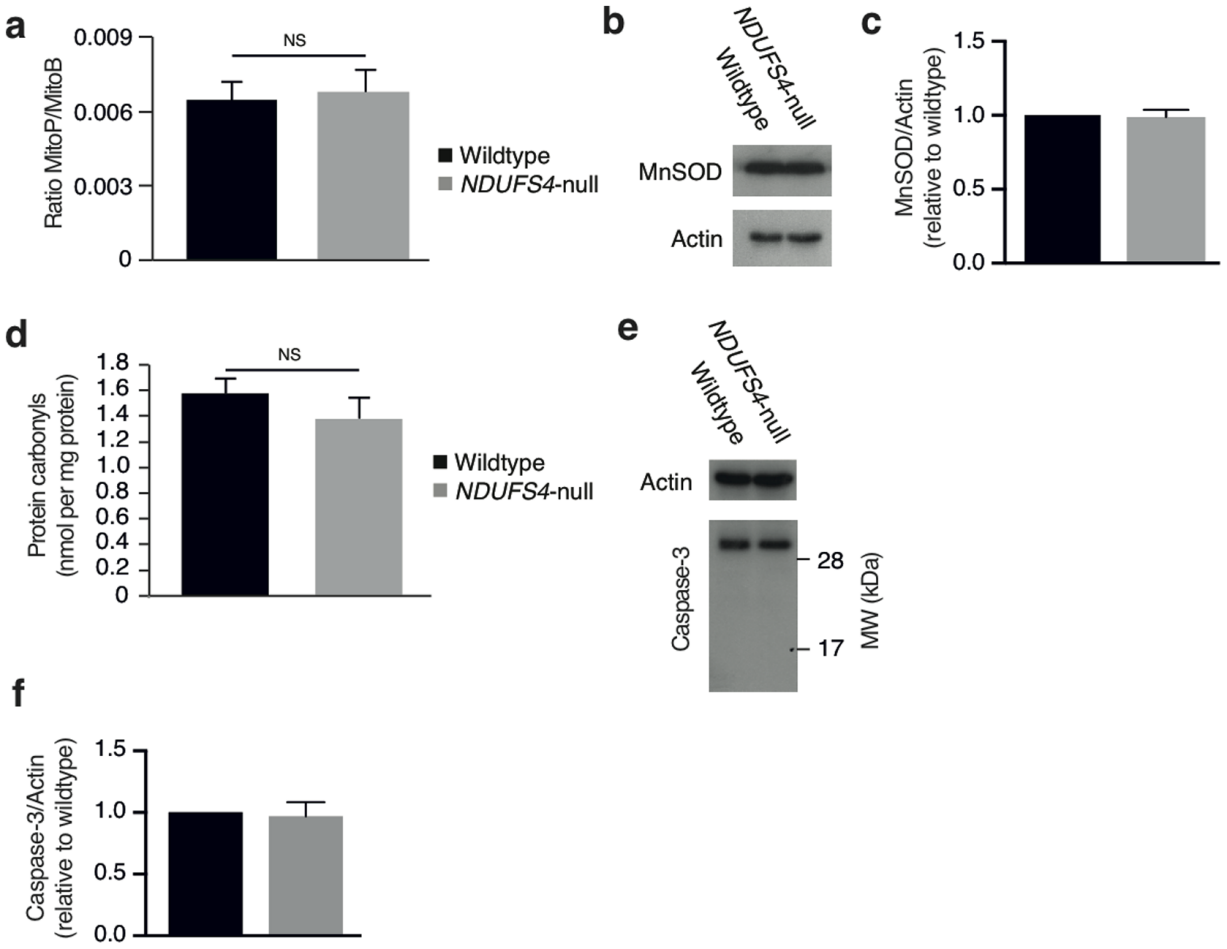
Assessment of mitochondrial ROS and apoptosis. a, *in vivo* mitochondrial hydrogen peroxide levels quantified using the ratiometric mass spectrometry probe MitoB. Hearts isolated from control and *Ndufs4*-null mice injected with MitoB are assessed by MS for MitoB and MitoP levels and the level of mitochondrial hydrogen peroxide are expressed as the ratio of MitoP to MitoB. **b**, Immunoblot determination and **c**, densitometry of MnSOD expression in control and *Ndufs4*-null hearts. **d**, Quantification of *in vivo* protein carbonylation in control and *Ndufs4*-null hearts. **e**, Immunoblot determination and **f**, densitometry of Caspase-3 expression and cleavage in control and *Ndufs4*-null hearts. n = 3–5.

Complex I is a major source of mitochondrial ROS, and cardiomyopathies are frequently associated with increased ROS levels [30]–[32]. A decrease in complex I activity could lead to an increase in mitochondrial NADH levels, which would in turn lead to an elevation of superoxide production by complex I and other sources [12]. Therefore, we next assessed whether *Ndufs4*-null mediated hypertrophic cardiomyopathy correlated with changes in mitochondrial ROS levels. To do so we used the recently developed MitoB probe for *in vivo* quantification of mitochondrial hydrogen peroxide production [27]. Interestingly, *Ndufs4*-null mouse hearts exhibited mitochondrial hydrogen peroxide levels indistinguishable from controls (Fig. 4a). Importantly, assessment of mitochondrial hydrogen peroxide *in vivo* is a reliable surrogate for levels of the proximal mitochondrial ROS, superoxide, since mitochondria have micromolar concentrations of MnSOD which reacts with superoxide extremely rapidly (*k*≈2.3×10^9^ M^−1^·s^−1^) to form mitochondrial hydrogen peroxide [12]. In parallel, we also measured the levels of the MnSOD in mild-type and *Ndufs4*-null mouse hearts to compare the endogenous superoxide dismutation capacity, finding levels to be comparable (Fig. 4b,c). In addition, measurement of protein carbonyls as an indication of oxidative damage showed that *Ndufs4*-null mouse hearts were similar to control hearts (Fig. 4d). Finally, we determined whether *Ndufs4* ablation initiated apoptosis by measuring cleavage and activation of caspase 3. While wild-type and *Ndufs4-*null mouse hearts had comparable levels of full length caspase 3, in both groups cleaved caspase 3 was undetectable (Fig. 4e,f). Together these findings indicated that the observed development of severe hypertrophic cardiomyopathy due to complex I disruption occurred independently of changes in mitochondrial hydrogen peroxide levels, oxidative damage, or apoptosis.

Here we have demonstrated that moderate and chronic disruption of mitochondrial complex I activity through heart-specific ablation of the Ndufs4 gene is sufficient to drive severe hypertrophic cardiomyopathy as determined by MRI. These gross pathological changes occur independently of alterations in mitochondrial hydrogen peroxide levels, oxidative damage, or apoptosis. Together these results suggest a primary role for complex I activity in driving the pathophysiological processes that lead to cardiomyopathy. Furthermore, complex I driven cardiomypathy occurs independently of aberrant ROS production and is thus primarily due to defective complex I activity that impacts on cardiac function by disrupting bioenergetic parameters in the heart.

## Supporting Information

Video S1 **Long-axis MRI view of heart function of control mouse hearts.**
(WMV)

Video S2 **Long-axis MRI of heart function of** ***Ndufs4*** **-null mouse hearts.**
(WMV)
